# SAMStat 2: quality control for next generation sequencing data

**DOI:** 10.1093/bioinformatics/btad019

**Published:** 2023-01-13

**Authors:** Timo Lassmann

**Affiliations:** Precision Health, Telethon Kids Institute, University of Western Australia, Perth, WA 6009, Australia

## Abstract

**Motivation:**

SAMStat is an efficient program to extract quality control metrics from fastq and SAM/BAM files. A distinguishing feature is that it displays sequence composition, base quality composition and mapping error profiles split by mapping quality. This allows users to rapidly identify reasons for poor mapping including the presence of untrimmed adapters or poor sequencing quality at individual read positions.

**Results:**

Here, we present a major update to SAMStat. The new version now supports paired-end and long-read data. Quality control plots are drawn using the ploty javascript library.

**Availability and implementation:**

The source code of SAMStat and code to reproduce the results are found here: https://github.com/timolassmann/samstat.

## 1 Introduction

The sequence alignment/map (SAM/BAM) format is the standard format for aligned short- and long-read data (Li *et al.*, 2009). To assess the quality of the mapping, basic statistics such as the fraction of mapped reads are commonly used. However, assessing why, or if, the mapping failed is far from trivial. Underlying causes for poor mapping rates may relate to the parameters given to the alignment algorithm, poor sequencing quality, failure to trim auxiliary sequences from reads prior to mapping or a combination thereof. A number of quality control tools have been developed to address this issue including fastQC (Andrews *et al.*, 2010), multiQC (Ewels *et al.*, 2016) and tools designed to display assay-specific metrics (Graubert *et al.*, 2021).

Our program SAMStat (Lassmann *et al.*, 2011) is unique by focusing on revealing differences between well and poorly mapped reads. We rely of the mapping quality scores reported by the aligner. When implemented correctly, these scores give the phred-scaled probability of an alignment being wrong [see supplementary material in Li *et al.* (2008)]. The presence of sequencing errors or simply lower base call qualities increases this probability. SAMStat takes advantage of the mapping qualities by displaying sequence composition, base qualities and error profiles along the read lengths in different mapping quality bins. This allows users to contrast the properties of the poorly mapped, or unmapped reads to confidently mapped reads and rapidly diagnose the root cause of poor mapping results. For example, poor mapping resulting from the presence of ‘N’s can become evident by contrasting the proportion of ‘N’ in confidently versus poorly or unmapped reads.

However, the previous version of SAMStat lacked support for newer sequencing technologies, paired-end protocols and was of limited utility when applied to long reads. Here, we present an updated and completely rewritten version of SAMStat to support a broader set of sequencing datasets.

## 2 Materials and methods

We have completely overhauled the C code for SAMStat from scratch. In contrast to the previous version, the new version is at least twice as fast and supports paired-end and long-read data. Furthermore, SAMStat can now generate QC reports for multiple input files in parallel using parallel programming threads. Under the hood, we now use the htslib API (Bonfield *et al.*, 2021) in lieu of samtools and unix pipes to read SAM/BAM files. A new parser based on a finite state automaton is used to read FASTA/FASTQ files. Long reads and long-read names are now fully supported. To improve the usefulness of quality control plots, we switched from static to interactive plots using the plotly javascript library. This allows for better visualization of long reads including the ability to zoom into regions of interest. In addition, we added functionality to examine the ends of reads and to view quality control metrics along the entire read length.

A SAMStat version 2 html report includes a basic statistics section, a base composition plot split by read fragment when multiple fragments are available, a length distribution plot and mismatch, insertion and deletion profile along the read length. Toggle buttons are used to switch between different quality control bins. Soft-clipped sequences are not displayed.

To assess the utility of SAMStat we downloaded three BAM files, some flagged as having QC issues, from the ENCODE project (The ENCODE Project Consortium, 2012).

## 3 Results

**Fig. 1.**
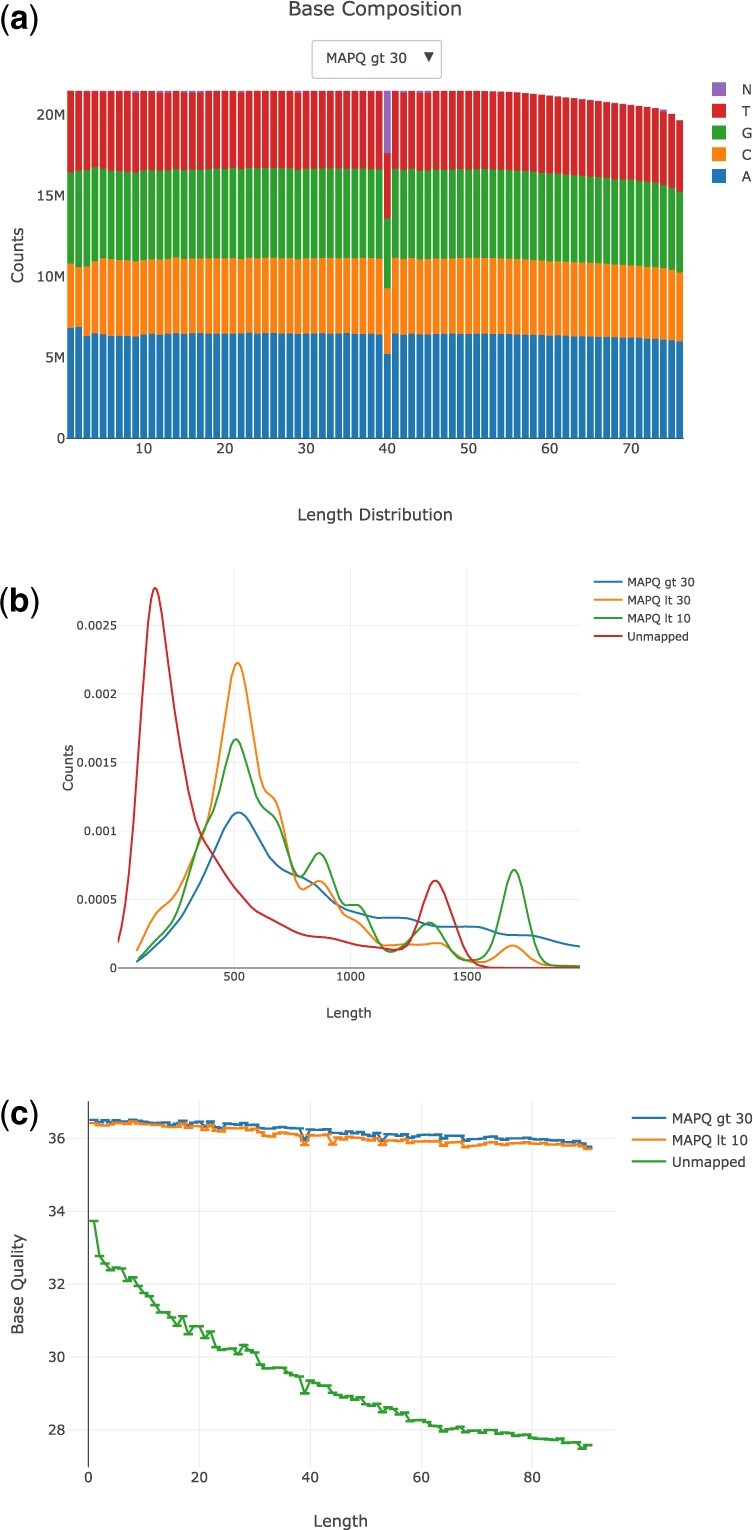
Selection of SAMStat QC plots. (**a**) Base composition of a polyA+ library. (**b**) Length distribution of a PacBio library, split by mapped and unmapped reads. (**c**) Mean base quality of a 10× single cell library

We ran SAMStat on standard polyA plus selected RNA-seq library, a long read RNA-seq library sequenced on the Oxford Nanopore MinION platform and a 10× single cell library (ENCODE file IDs: ENCFF039WAK, ENCFF050BNR and ENCFF596PVI, respectively). Figure 1 shows a selection of the QC plots produced by SAMStat for the three different libraries. While there are no major QC issues with the standard RNA-seq library, we can see that position 40 in the reads contains a high number of N’s (Fig. 1a). By plotting the read length distribution of the Nanopore data, we can clearly see that unaligned reads have a bimodal length distribution (red line, one peak at 165 nt and another at 1360 nt). In contrast, the majority of aligned reads are longer than 250 nt, and reads in different mapping quality bins have distinct length profiles (Fig. 1b). Unmapped reads of the 10× library have a noticeable lower base call quality compared to the aligned reads (Fig. 1c). These examples illustrate how SAMStat can be used in existing data processing workflows to provide additional quality control information and to aid in diagnosing the cause of poor mapping results.

## 4 Conclusion

We present a new version of our quality control tool SAMStat with support for a broader range of sequencing protocols. SAMStat complements existing tools by providing a deeper view of read-mapping issues.
